# Hybrid Wireless Fingerprint Indoor Localization Method Based on a Convolutional Neural Network

**DOI:** 10.3390/s19204597

**Published:** 2019-10-22

**Authors:** Zhenyu Liu, Bin Dai, Xiang Wan, Xueyi Li

**Affiliations:** School of Information Engineering, Guangdong University of Technology, Guangzhou 510006, China; zhenyuliu@gdut.edu.cn (Z.L.); wx1tching@163.com (X.W.); leexueyi@gdut.edu.cn (X.L.)

**Keywords:** indoor location, convolutional neural network, fingerprint, WiFi, RSSI

## Abstract

In the indoor location field, the quality of received-signal-strength-indicator (RSSI) fingerprints plays a key role in the performance of indoor location services. However, changes in an indoor environment may lead to the decline of location accuracy. This paper presents a localization method employing a Hybrid Wireless fingerprint (HW-fingerprint) based on a convolutional neural network (CNN). In the proposed scheme, the Ratio fingerprint was constructed by calculating the ratio of different RSSIs from important contribution access points (APs). The HW-fingerprint combined the Ratio fingerprint and the RSSI to enhance the expression of indoor environment characteristics. Moreover, a CNN architecture was constructed to learn important features from the complex HW-fingerprint for indoor locations. In the experiment, the HW-fingerprint was tested in an actual indoor scene for 15 days. Results showed that the average daily location accuracy of the K-Nearest Neighbor (KNN), Support Vector Machines (SVMs), and CNN was improved by 3.39%, 8.03% and 9.03%, respectively, when using the HW-fingerprint. In addition, the deep-learning method was 4.19% and 16.37% higher than SVM and KNN in average daily location accuracy, respectively.

## 1. Introduction

In the last few decades, location-based services (LBS) have become an integral part of daily life. Nowadays, satellite location systems have poor accuracy indoors because indoor environments have more obstacles that cause severe attenuation to satellite signals [1]. To obtain a satisfying real-time indoor location, many methods have been proposed. An indoor location method based on Radio Frequency IDentification (RFID) was proposed in [2,3]. However, the method has the following disadvantages: anti-interference is poor, and the biggest problem is that, when the mobile terminal does not actively scan the RFID tag, localization cannot be implemented. In recent years, Ultrawideband (UWB) [4] and infrared [5] have achieved high precision in indoor location applications and attracted the attention of many researchers. However, the difficulty of implementation and relative high hardware costs restrict its practical applications.

In many RSSI-based indoor localization technologies, location based on WiFi has become popular because of the widespread use of wireless local-area networks (WLANs) [6]. The WiFi-based indoor location method is divided into two types, one based on a propagation model and another based on the RSSI fingerprint. The method based on the propagation model uses the time of arrival (TOA) [7,8] of the signal between nodes or the angle of arrival (AOA) of the signal [9] to determine the position coordinates. The Distance Vector-Hop (DV-Hop) algorithm is a very frequently used algorithm for Wireless Sensor Network(WSN). DV-Hop estimates the distance through the hop count between nodes in which the value of the hop count is discrete; thus, there is the serious consequence that some nodes have the same estimated distance when their hop count with respect to identical nodes is equal [10]. The propagation model requires the location of known signal nodes, and the existence of the propagation model of the wireless signal in an indoor environment is complicated, so it is difficult to have available location accuracy [11]. The fingerprint-based location method is a common method because of its simple implementation and acceptable accuracy [12].

The key issue of indoor location based on WiFi is the change of RSSI over time. RSSI changes caused by transient disturbances, such as moving objects and doors opening/closing, can be eliminated by adding RSSI fingerprint samples. However, long-term changes such as weather changes, which cause water-density differences in the environment, should be taken into account. As mentioned in [13], moisture absorbs WiFi signals and causes them to attenuate. In indoor locations, the quality of a fingerprint database heavily determines the effect of indoor location services [14]. Many methods for improving the expression of RSSI fingerprints on indoor-environment features have been proposed. In [15], a preprocessing method was proposed that involves deleting useless access points (APs) from the fingerprint database. At present, WiFi devices mainly use the 2.4 GHz frequency band. When moisture in the air changes, the WiFi signal has different attenuation. However, the influence of moisture on indoor air is rarely considered.

In order to have satisfying indoor location accuracy, in [16], K-Nearest Neighbor (KNN) was used to match similar RSSI sequences for locations from the fingerprint database, but KNN is sensitive to data noise, which makes location results prone to errors. In order to enhance location reliability, a Bayesian method for solving indoor positioning problems was proposed in [17,18]; the complex distribution of indoor RSSI data makes the Bayesian method unable to fit RSSI data well. In [19], a positioning system was proposed based on Support Vector Machines (SVMs) that treated a positioning task as a classification problem. This machine-learning method could achieve considerable indoor positioning accuracy, but the above algorithms usually learn shallow features of data. A complex and varied indoor environment makes indoor WiFi signal data very complicated, which leads to the machine-learning method not being able to extract all reliable features from the complex RSSI fingerprint.

Recently, deep learning has received a lot of attention due to high accuracy in the case of a large amount of training data [20,21,22]. Compared to the KNN and SVM techniques, there are deep-learning attempts to learn high-level features from data in an incremental manner, which decreases demand for domain expertise and specific-feature extraction [23]. Although the deep-learning algorithm is costly in the offline phase due to a large number of parameters, time costs in the online phase are smaller, which is why the deep-learning method has attracted so much attention [24]. A method for fingerprint location using a Deep Neural Network (DNN) and Wi-Fi was proposed in [25] that improved accuracy by using a hidden Markov model (HMM). HMM prediction is based on a previous position obtained through the DNN that could have error accumulation [26]. The authors of [27] put forth a dictionary learning framework for fingerprinting indoor locations using GSM, WiFi, and other sensor measurements, but this leads to more complicated implementation. DeepFi [28] and ConFi [29] proposed a method of creating a fingerprint using the channel-state information (CSI) of a Wi-Fi signal. However, CSI information in these methods is obtained by specific hardware. Deep Belief Networks (DBN) [30] are also used for indoor positioning. This technology is based on specific UWB beacons but has high implementation costs.

The Convolutional Neural Network (CNN) method was proposed in [31], where the problem of indoor positioning is transformed into image classification by reconstructing the RSSI fingerprint to a grayscale image. However, this does not consider environment changes over time. In [32], a tracking and positioning method based on CNN and Bluetooth was proposed where the author transferred the problem of indoor locations into image identification and built pixel grayscale image according to real environments and known Bluetooth nodes. In an actual environment, it is difficult to investigate the location of each AP, and artificially deploying APs leads to increased location costs.

As mentioned above, some machine learning was proposed to solve the problem of indoor positioning, but it is difficult for general machine-learning methods to learn deep features. In this work, we used a CNN to turn the problem of indoor positioning into a classification problem. CNN has also been used in some studies, but the literature [32] needed to know the physical layout of the indoor environment, and [31] did not consider the impact of time changes on indoor positioning. Therefore, we propose a HW-fingerprint approach that does not require an understanding of the indoor-environment layout and takes the effects of time changes into account.

The main contributions of this work are as follows:

- HW-fingerprint is proposed. The ratio relationship between APs was combined with the RSSI fingerprint;
- CNN architecture was constructed to effectively learn characteristics from the HW-fingerprint;
- Different CNN architectures were tested to find the best location model;
- The proposed method was verified with 15 days of collected data in the actual environment. The tested algorithm had better location results when using the HW-fingerprint.

The rest of this article is organized as follows. Section 2 describes the proposed method in detail. Section 3 shows the experiment results and discusses the proposed work. Finally, conclusions are given in Section 4.

## 2. Proposed Methods

**Figure 1:**
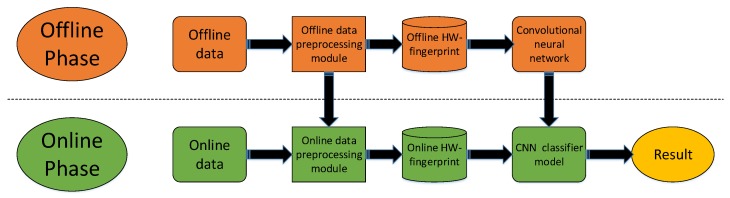
Proposed system architecture.

Figure 1 shows the proposed methods, where we constructed the HW-fingerprint both in an offline and online phase. The deep-learning method was used to learn the features of the HW-fingerprint and predict the indoor location. In the offline data-processing module, we could obtain the Media Access Control (MAC) address sequence of the relevant AP that was used to construct the ratio fingerprint, and also to match and construct the Ratio fingerprint corresponding to the online HW-fingerprint. At the same time, information about the range of RSSI and the Ratio fingerprint obtained in the offline data-processing module was also used to standardize the online HW-fingerprint. In the offline phase, we trained a CNN model for indoor locations, and used this model to predict the location of online data in the online phase.

Subsequently, we introduce the location method in detail. First the reasons for building a HW-fingerprint are introduced. Second, the processes of building the HW-fingerprint in offline and online phases are described. Third, reasons of treating the location problem as image classification are described. Finally, details of the CNN location architecture are presented.

### 2.1. Analysis of HW-Fingerprint Construction

In this subsection, we analyze RSSI changes in the environment and describe the ideas for the construction of the Ratio fingerprint. As we know, currently used WiFi devices mainly use the 2.4 GHz band. A 2.4 GHz WiFi signal is severely attenuated in water (microwaves are heated up by water molecules absorbing the energy resonance of this band). If the water density in the air changes, the WiFi signal has different attenuation.

**Figure 2:**
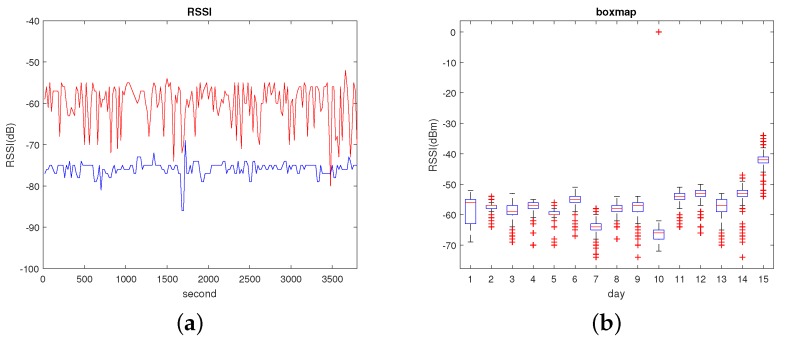
Received-signal-strength-indicator (RSSI) changes. (**a**) Changes of the RSSI of two Access Points (APs) in a short period; (**b**) box-diagram description of RSSI AP changes in 15 days.

Shown in Figure 2a are changes in RSSI values of two APs collected by same reference point (RP). RSSI distribution was relatively stable in the short term. Therefore, good location results can often be achieved in the short term. As we know, indoor environments have more unstable factors than outdoor environments. There is much disturbance in an indoor environment, such as instantly moving targets or someone temporarily standing next to the AP and blocking the WIFI signal. instant RSSI positioning measurements are not sufficient as fingerprints for location, and these temporary interferences can be solved by collecting more samples.

In Figure 2b, a box diagram of RSSI changes of APs in the next 15 days is described. RSSI distribution is very different in different days. This is a reason why the method based on propagation-model positioning needs to update the propagation model in time. Table 1 shows the weather for five days; it can be seen that the weather is different every day, whether in terms of temperature or weather conditions. Therefore, the RSSI becomes unstable when the environment changes, so here we mainly considered the change of daily RSSI values due to the change of water molecular density in the air.

In the case of WiFi devices using a 2.4 GHz frequency band, if water density in the air changes, the WiFi signal has different attenuation, but RSSI attenuation caused by different water molecular densities per day means that the RSSI ratio between different APs is relatively stable when all AP in same air humidity environment at the same attenuation rate. According to previous analysis, this gave us the idea of constructing the corresponding Ratio fingerprint by calculating the ratio of RSSI between different APs. The constructed Ratio fingerprint was considered to be added into the RSSI fingerprint to construct the proposed HW-fingerprint, which could make up for the RSSI fingerprint not being able to express indoor environments well.

### 2.2. Acquisition of Offline HW-Fingerprint

**Figure 3:**
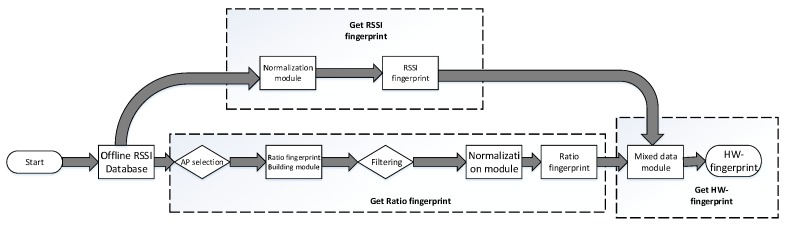
Flowchart of building Hybrid Wireless (HW)-fingerprint.

In this subsection, we divide the process of obtaining the HW-fingerprint offline into three parts as indicated by the dashed box in Figure 3. In order to obtain the HW-fingerprint, we copied the offline RSSI database, one for obtaining the offline RSSI fingerprint and the other for obtaining the Ratio fingerprint, and finally merged them to obtain the final HW-fingerprint.

#### 2.2.1. Acquisition of RSSI Fingerprint

In order to obtain RSSI fingerprints, the offline RSSI database was processed by a normalization module; the detailed process is as follows.

$RSSI_{m}^{i,j}$ denotes the offline RSSI database, *m* is the *m*-th RP (where m=1,…,M and *M* are the total RPs set in the indoor environment), *i* is the *i*-th measurement fingerprint in *m*-th RP (where i=1,…,I and *I* are the total number of measurements in *m*-th RP), and *j* denotes the *j*-th AP (where j=1,…,J and *J* are the total APs that could be collected in the environment). Let the maximum RSSI in $RSSI_{m}^{i,j}$ be $RSSI_{MAX}$, and the minimum RSSI be $RSSI_{MIN}$. In the normalization module, the RSSI fingerprint could be constructed by standardizing $RSSI_{m}^{i,j}$ as the following equation:

(1) $$RSSI_{m,new}^{i,j}=\frac{RSSI_{m}^{i,j}-RSSI_{MIN}}{RSSI_{MAX}-RSSI_{MIN}}$$

#### 2.2.2. Acquisition of Ratio Fingerprint

To obtain the Ratio fingerprint, the specific implementation was divided into the following steps:

(1) In the AP selection module, the contribution weight of every AP in the initial RSSI fingerprint needed be calculated. $Number_{j}$ denotes the number of collection times in the *j*-th AP (where j=1,…,J and *J* are the total APs that could be collected in the environment); the total number of RSSI fingerprint samples collected in the indoor environment is $Number_{total}$. Then, the *j*-th AP’s contribution weight was calculated with the following equation:

(2) $$W_{j}=\frac{Number_{j}}{Number_{total}}$$

where $W_{j}$ is the contribution weight of *j*-th AP.

After obtaining the contribution weight of each AP, we set a minimum weight threshold. We then selected the APs of which the weight was above the minimum threshold and obtained its corresponding MAC sequence and RSSIs.

(2) In the ratio-fingerprint building module, we could obtain the initial Ratio fingerprint. Let the MAC address sequence of the selected AP be $MAC_{importent}=\left\{MAC_{1},MAC_{2},\ldots ,MAC_{V}\right\}$, where *V* belong to 1,…,M and is less than *M*. Their corresponding RSSI is $RSSI_{importent}=\left\{RSSI_{1},RSSI_{2},\ldots ,RSSI_{V}\right\}$. According to the corresponding RSSI data of the AP, the Ratio fingerprint was constructed by the following equation:

(3) $$Ratio_{p}=\left\{\begin{array}{cc} \frac{RSSI_{K}}{RSSI_{I}}, & \mathrm{if}\;RSSI_{K},RSSI_{I}\neq 0,\;\mathrm{and}\;1\leq K<I\leq V\\ 0, & \mathrm{if}\;RSSI_{K},RSSI_{I}\;\mathrm{Both}\;\mathrm{are}\;0\;\mathrm{or}\;\mathrm{have}\;a\;0 \end{array}\right.$$

where $1\leq p\leq \sum_{1}^{v-1}x$, and x belongs to 1≤p≤V−1. We could then obtain the Ratio fingerprint.

(3) The filter was set to filter outliers of the initial Ratio fingerprint. In the filter module, the ratio with 0 should not be counted in the total. The quartile of the Ratio fingerprint was obtained by the box-plot analysis method. The outliers in the Ratio fingerprint were defined as:

(4) $$\begin{array}{c} \left\{\begin{array}{c} ErrorValue<Q_{l}-1.5*IQL\\ ErrorValue>Q_{u}-1.5*IQL \end{array}\right. \end{array}$$

where $Q_{l}$ is the lower quartile, $Q_{u}$ is the upper quartile, and IQL is the quartile range. We filtered out the outlier according to Formula (4) and filled the exception element with a value of 0.

(4) In the normalization module, we normalized the Ratio fingerprint: we set the maximum ratio as $Ratio_{MAX}=Q_{u}+1.5*IQL$, and the minimum ratio as $Ratio_{MIN}=Q_{l}-1.5*IQL$; then, the normalization formula for the Ratio fingerprint was:

(5) $$Ratio_{p}^{new}=\frac{Ratio_{p}-Ratio_{MIN}}{Ratio_{MAX}-Ratio_{MIN}}$$

where $p=\sum_{1}^{v-1}x$.

#### 2.2.3. Acquisition of HW-Fingerprint

To obtain the HW-fingerprint, the Ratio fingerprint in the mixed-data module had to be combined with the RSSI fingerprint. Let one of the RSSI fingerprint samples be $RSSI=(RSSI_{1},RSSI_{2},\ldots ,RSSI_{J})$; *J* is the total that could be detected in indoor environments. Then, the correspondingly constructed ratio-fingerprint sequence is $Ratio=(Ratio_{1},Ratio_{2},\ldots ,Ratio_{p})$, $p=\sum_{1}^{V-1}x$. Its HW-fingerprint sequence is $HW=(RSSI_{1},RSSI_{2},\ldots ,RSSI_{J},Ratio_{1},Ratio_{2},\ldots ,Ratio_{p})$.

### 2.3. Acquisition of Online HW-Fingerprint

Online location RSSI data are transferred to the HW-fingerprint like the offline HW-fingerprint. With the processing foundation of obtaining the HW-fingerprint in offline phase, some steps can be utilized to keep the HW-fingerprint obtained in the offline and online phases consistent. The implementation process specifically includes the following steps:

Step 1: Matching online RSSI data based on the MAC sequence that constructs the initial RSSI fingerprint and normalizes the online RSSI fingerprint according to the upper and lower limits that were set in Section 2.2.1.

Step 2: According to the $MAC_{importent}=\left\{MAC_{1},MAC_{2},\ldots ,MAC_{V}\right\}$ sequence from the offline phase, we matched its associated online RSSIs. Then, we constructed the online Ratio fingerprint with the method of obtaining the Ratio fingerprint in Section 2.2.2.

Step 3: According to Section 2.2.3, we combined the RSSI fingerprint and ratio-fingerprint sequences to form the online HW-fingerprint.

### 2.4. Indoor-Location Analysis and Image Classification

**Figure 4:**
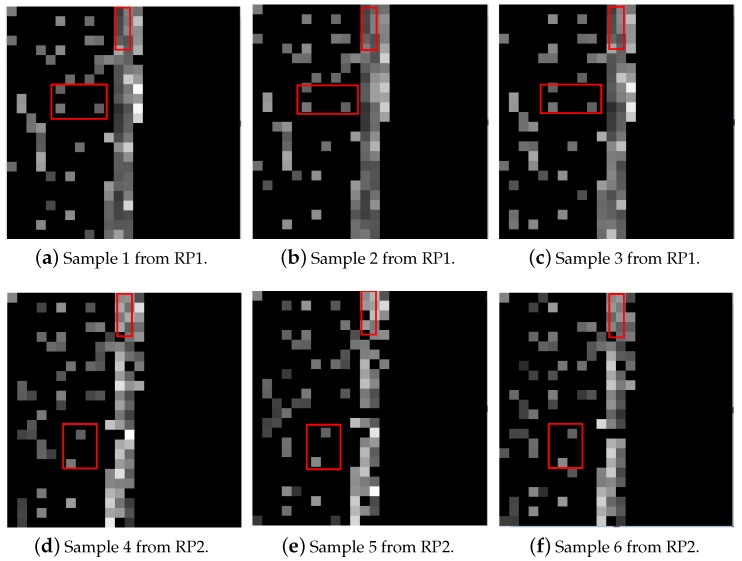
Grayscale images constructed by HW-fingerprint. (**a**–**c**) from RP1; (**d**–**f**) from RP2.

Since a CNN has strong feature-extraction capabilities in image classification, this helped us to translate the problem of indoor location into image classification. According to the HW-fingerprint obtained in Section 2.2, the RSSI fingerprint was compressed into a distribution of 0–1 by $RSSI_{MAX}$ and $RSSI_{MIN}$, and the Ratio fingerprint was normalized to the range of 0–1 through the outlier boundary of ErrorValue. In the image, the brightness of the grayscale image was 0–255. Finally, we reshape the vector into a matrix. Considering the multiplication of 255 for each feature of the HW-fingerprint, we could obtain the brightness distribution of the HW-fingerprint in the image. These luminance distributions give us a visual representation of the HW-fingerprint. As shown in Figure 4a, the sparse part of the white- and gray-pixel distribution on the left side was constructed by the RSSI fingerprint, while the dense part of the white pixel in the middle was constructed by the Ratio fingerprint, and the black part on the right side was the space reserved for the possible Ratio fingerprint. In the Ratio fingerprint, the dimension of the ratio fingerprint was determined by the number of selected APs.

We set up some RPs in our laboratory that collected RSSIs for the following experiment. The specific settings are described in detail in the subsequent experiment sections. As shown in Figure 4, grayscales a, b, and c were visualized by different samples from RP1. The RP is a collection point of the RSSI fingerprint. Generally, an indoor environment is divided into multiple subareas, and collection points are generally set at the center of the subareas. Grayscales e, d, and f in Figure 4 are visualized by different samples from RP2, and RP2 is another collection point, different from RP1. In Figure 4, we marked some regions with a rectangle in six grayscales, where it can been seen that samples from the same RP had similar brightness and pixel distribution at the marked locations, while samples from different RPs had different brightness and pixel distribution at the marked regions; this visual difference enlightens us to treat the problem of indoor location as a problem of image classification.

Similarly, the HW-fingerprint contains a lot of noise. In Figure 4, it can be seen that even samples from the same RP still have differences, which reflects the complex and varied characteristics of indoor environments. In order to solve this problem, in the following content, we introduce the CNN location model we constructed. The CNN was used to learn useful features from the HW-fingerprint with much noise and to determine user position.

### 2.5. Deep-Learning Location Model

DNNs, Recurrent Neural Networks (RNN), and CNNs are commonly used in classification. The hidden layer neurons of a DNN are connected with all neuronal inputs from the previous layer, resulting in a large number of parameters to be learned, so it becomes difficult to obtain a suitable model when the data dimension is high. Both CNNs and RNNs are improved networks based on DNN. RNNs are mostly used to deal with time-series problems and it is often used in the field of natural-language processing. CNNs capture the relationship between local regions from a spatial perspective, and is often used in computer vision to classify images and achieve better results in image classification. Since a CNN has strong feature-extraction capabilities in image classification, this motivated us to translate the problem of indoor location into image classification.

The CNN location model mainly consists of the following parts:

#### 2.5.1. Convolution Layer

The convolutional layer can extract feature maps within local regions in the previous layer’s feature maps with linear convolutional filters followed by nonlinear activation functions. Denote $\theta_{i}^{l}$ i as the *i*-th feature map in layer of the CNN, which is defined as:

(6) $$\begin{array}{c} \theta_{i}^{L}=\delta \left(\sum_{m\in S_{L-1}}w_{im}^{L}*\theta_{m}^{L-1}+b_{i}^{L}\right) \end{array}$$

where δ is the Rectified Linear Units (ReLUs) function, $b_{i}^{L}$ is the bias of the *i*-th feature map in layer *L*, $S_{L-1}$ is the set of feature maps in layer L−1 connected to the current feature map, and $w_{im}^{L}$ is the convolutional kernel to generate the *i*-th feature map in layer *L*, which is the same for different *m* due to local weights sharing. The convolution operation can obtain the shift invariance of input data and extract robust features.

#### 2.5.2. Pooling Layer

Pooling layer is a downsampling layer that downsamples the outputs of the previous convolutional layer. It can reduce computational complexity by reducing the dimension of tensors. We chose the max-pooling function, which selects the maximum value of those covered within the currently chosen pooling window.

#### 2.5.3. Fully Connected Layer and Output Layer

For the fully connected layer, we utilized a basic neural network with a hidden layer to train the output data after all the convolutional and subsampling layers. Moreover, a softmax layer was employed as an output layer to calculate the output label. The softmax layer is defined as:

(7) $$S_{i}=\frac{e^{i}}{\sum_{j}^{a}e^{j}}$$

where $S_{i}$ is the probability that the input data belong to the ith location, and *a* is the total number of location tags.

#### 2.5.4. ReLUs

In order to reduce the occurrence of overfitting, the Rule layer was adopted to CNN as an activation function in the convolutional and fully connected layers. Compared to traditional neural-network activation functions, such as logic functions (logistic sigmoid), tanh, and other hyperbolic functions, Rectified Linear Units (ReLUs) function have the following advantages: Firstly, the principle of biological and related brain research shows that the information coding of biological neurons is usually scattered and sparse. Secondly, more efficient gradient descent and backpropagation that avoids gradient explosion and gradient disappearance. Finally, simplification of the calculation process, where there is no influence of other complex activation functions such as exponential function. At the same time, dispersion of activity decreases the overall computational cost of the neural network. A ReLU is defined as

(8) $$f_{Relu}\left(x\right)=\left\{\begin{array}{cc} x, & \mathrm{if}\;x\;>\;0\\ 0, & \mathrm{otherwise} \end{array}\right.$$

### 2.6. Training Process

In training process, the training parameters shown in Table 2, during the training process were training epoch, batch size, and learning rate, and they were set as 20, 50, and 0.001, respectively. In addition, dropout technology was added in the fully connected layer to prevent network overfitting. The dropout parameter in the network was set to 0.5, which means that neurons in the fully connected layer were closed with a probability of 0.5, so that they did not participate in any calculations and in the update of the weights.

**Figure 5:**
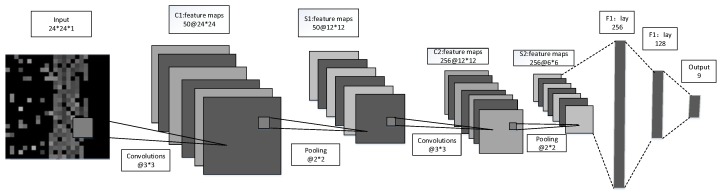
Convolutional neural network (CNN) architecture.

The proposed CNN architecture is shown in Figure 5. In order to learn the location features from the HW-fingerprint, the learning process of the proposed CNN was as follows: The images were convenient for the CNN to process in its convolution and pooling layers. For each input image in the first convolutional and pooling layer, we employed 50 convolutional filters with 3 * 3 size to obtain the same number of feature maps with 24 * 24 size that could extract different characteristics. Simultaneously, the same number of feature maps with 12 * 12 size could be obtained by pooling sized 2 * 2. Then, by implementing one more convolutional and pooling layer, as shown in Figure 5, we obtained 256 feature maps sized 6 * 6. We reshaped them to a vector and filled them in the fully connected (FC) and SoftMax layers to obtain the probability that the fingerprint belonged to each region.

## 3. Experiment Results and Discussion

### 3.1. Experiment Setup

**Figure 6:**
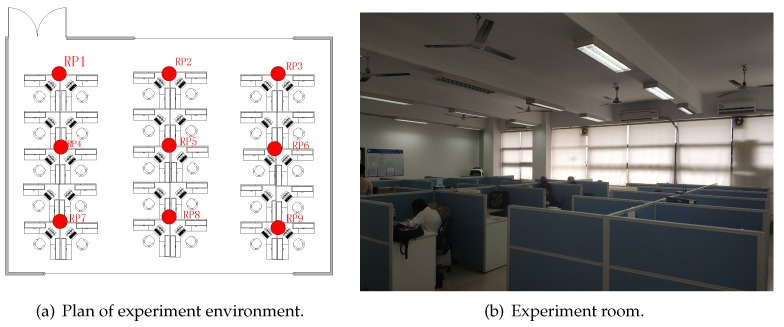
Experiment environment diagram.

In order to collect the necessary evaluation data, the proposed method was deployed in Lab 505 of the Engineering Facility Building No.1 of Guangdong University of Technology. As shown in Figure 6, the laboratory area is 12.5 × 10 m, each RP was set in the center of the 3 × 3 m square area, as shown in Figure 6a with a red point, and a total of 9 RPs were set. In the test environment, 258 unknown APs were detected by our mobile devices. Thousands of RSSI samples were collected on the first day by nine RPs. Data collection was performed for half a month.

The WiFi collector application was implemented to collect surrounding WiFi information, and the program recorded the MAC, RSSI, and timestamp of every sample. Construction and training of the CNN model was based on Google’s open-source deep-learning framework of TensorFlow (version 1.8) [33]. The data from the first day were used for training, and data from the subsequent days were used for testing.

In the experiment, we used the HW-fingerprint constructed by the RSSI data collected on the first day as the training data. The data were continuously collected for testing in the RPs where the training data were collected in the subsequent time. In order to compare the experiment results, we defined the accuracy of the prediction. Let $NUM_{correct}^{Di}$ be the number of samples predicted to be correct on the *i*-th day, and $NUM_{totol}^{Di}$ is the total test sample data on *i*-th day. Then, the predicted accuracy of the *i*-th day was given by the following formula:

(9) $$Accuracy^{Di}=\frac{NUM_{correct}^{Di}}{NUM_{totol}^{Di}}\times 100\%$$

### 3.2. Threshold Impact on Location

**Figure 7:**
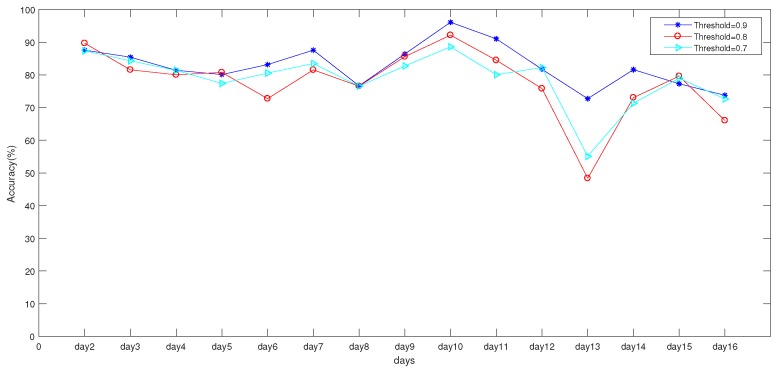
Accuracy of different thresholds.

In this subsection, the effects of different thresholds of the constructed HW-fingerprint are analyzed. All measurable APs in the indoor environment were used to select the AP with an important contribution to construct the Ratio fingerprint in the offline phase. For this reason, three thresholds were set to select APs with important contributions. When the threshold was low, it is worth noting that most of the APs were selected for ratio-fingerprint construction, and some unimportant APs were also selected. In order to obtain a suitable threshold, three sets of HW-fingerprints were constructed from thresholds of 0.7, 0.8, and 0.9, respectively. Figure 7 shows the three sets of HW-fingerprint prediction accuracy with the CNN for 15 days. It can be seen that, when the threshold was set to 0.9, results were better than with the other datasets.

To further analyze the appropriate thresholds, result statistics of the three datasets are shown in Table 3. When the threshold was set to 0.9, average accuracy was significantly better than with the two other thresholds. As shown in Figure 7, in the 13th day, when the threshold was set to 0.9, accuracy was not as good as during the other days, but it was better than the other two datasets. The main reason was that, on the 13th day, there was long-term power outage in experimental area that directly caused changes in the indoor environment.

### 3.3. Influence of Different CNN Structures on Location

AlexNet uses very large convolution kernels, such as 11∗11 and 5∗5. The idea is that the larger the convolution kernel is, the larger the receptive field and the picture information seen are. That being said, a large convolution kernel can lead to a surge in computational complexity, which is not conducive to increasing model depth and reducing computational performance.

**Figure 8:**
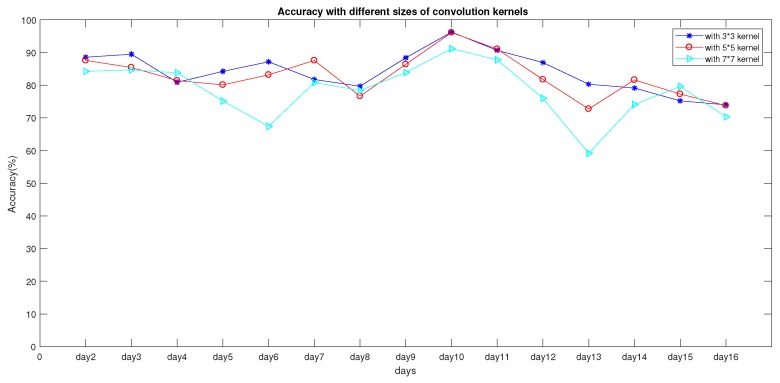
Impact of different thresholds on accuracy.

In this section, the influences of using different convolution-kernel sizes in a CNN were researched, as shown in Figure 8. When the convolution kernel was set to 3∗3, accuracy in subsequent location prediction was better than in the case where the convolution kernel was set to 5∗5 and 7∗7. As shown in Table 4, when kernel size was set to 3∗3, average accuracy was higher than in the others, and variance was lower than in the others.

In image recognition, a pooling layer is widely used in convolutional neural networks. It is used for feature-dimensionality reduction, compressing the number of data and parameters, reducing overfitting, and improving the fault tolerance of the model. However, we do not know whether the max-pooling layer in indoor positioning has an effect on indoor location.

**Figure 9:**
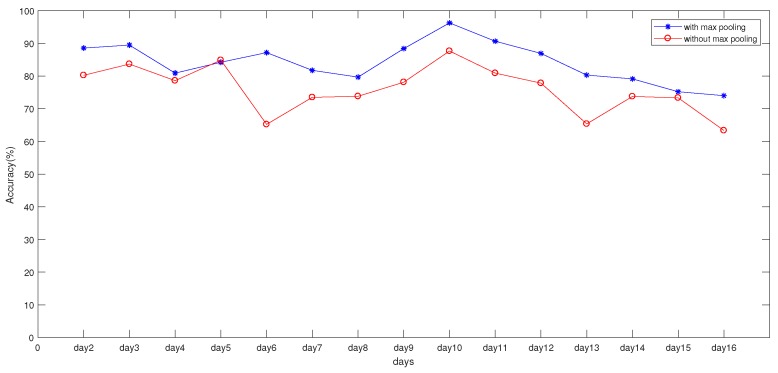
Comparison between experiment results with and without Max pooling.

**Figure 10:**
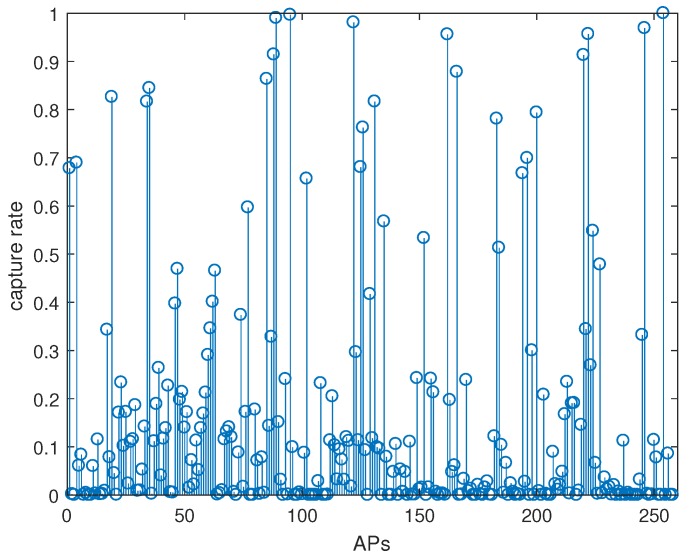
AP capture rate of RP point.

In order to compare the influence of the pooling layer on positioning, we constructed a full convolutional-neural-network structure. In the constructed full convolutional neural network, the original pooling layer was removed based on the previously constructed convolutional neural network. To achieve a better results, a convolutional layer was added. As shown in Figure 9, when the max-pooling layer was added, location accuracy was significantly higher than when the max-pooling layer was not added. Figure 10 shows the AP capture rate in an RP, which is the value of $Dect_{times}^{CR}$ to $ALL_{num}^{CR}$, where $Dect_{times}^{CR}$ means the times that it can be detected in RP, and $ALL_{num}^{CR}$ is the total number of fingerprint samples collected by the RP. It can be seen that many APs had a capture rate of less than 50%, which means that there was a lot of noise data in the HW-fingerprint, which also proved that the max-pooling layer had a filtering effect on the noise data.

### 3.4. Experiment Comparison of HW-Fingerprint

**Figure 11:**
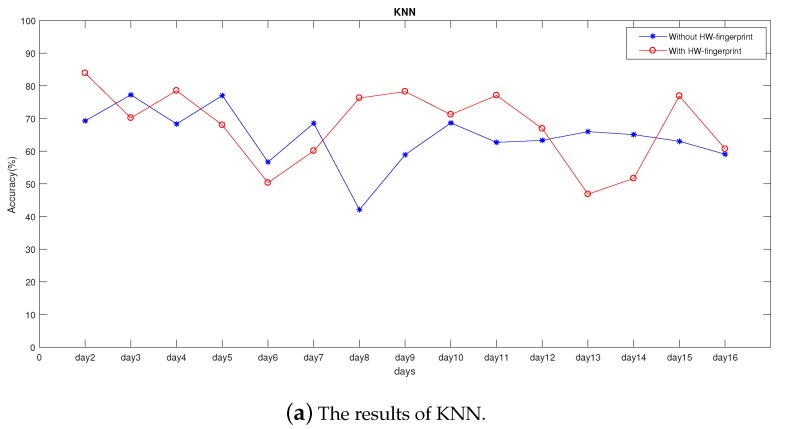

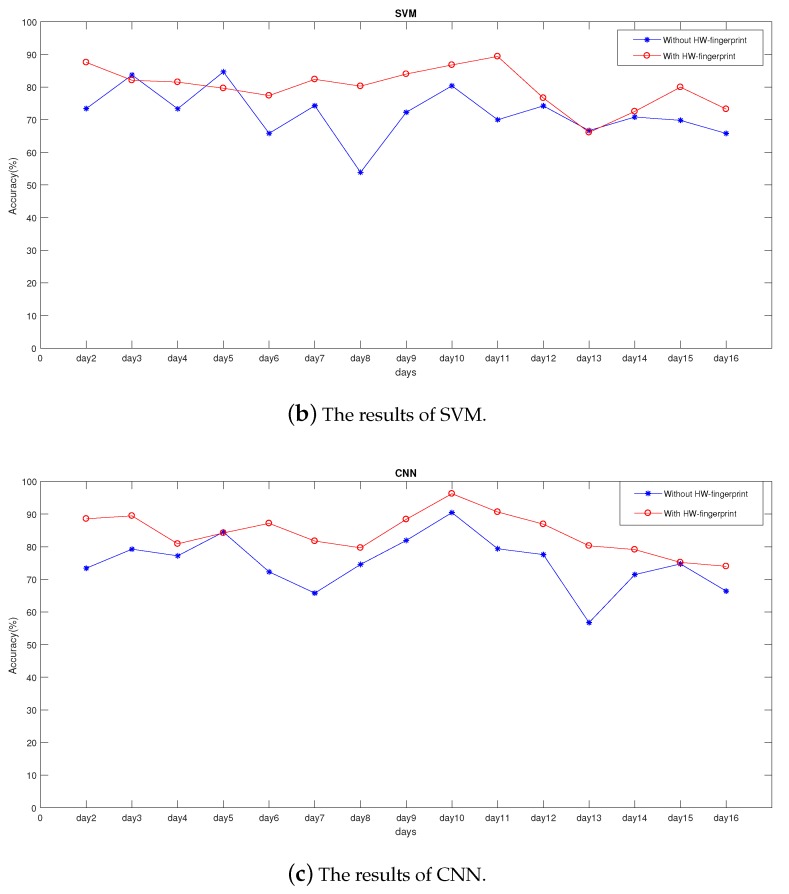
The results of experiment with and without HW-fingerprint.

In order to explore the rationality of the HW-fingerprint, we tested several other common machine-learning methods. Figure 11a–c gives the predictions of KNN, SVM, and CNN, respectively, in the case with and without the HW-fingerprint. Table 5 shows the average daily prediction accuracy of KNN, SVM, and CNN in the case with and without the use of the proposed HW-fingerprint. In the case with the HW-fingerprint, prediction accuracy was 67.79%, 79.97%, and 84.17% per day by KNN, SVM, and CNN, respectively, and in the case without HW-fingerprint, average daily location accuracy was 64.39%, 71.94% and 75.13%. It can be seen that, when using our HW-fingerprint, overall prediction accuracy was better than in cases without the HW-fingerprint. The average daily location accuracy of KNN, SVM, and CNN was improved by 3.39%, 8.03%, and 9.03%, respectively.

As shown in Figure 11a, only eight out of 15 days, or only half of the predictions, were better than those without the HW-fingerprint. Here, we analyzed the reasons. One was that KNN has no learning process. When predicting, it traverses all training data to select the K-nearest samples to determine the most probable prediction. As mentioned above, the value of K has a directly influence on prediction results and in order to simulate realistic predictions, we set the K value to 1 instead of tuning the value of K for better results. Another reason is that the HW-fingerprint dimension was higher than that of the RSSI fingerprint, and the KNN calculated the sample distance. As the dimension increased, correlation between the nearest sample and the predicted sample selected according to the distance decreased.

To further analyze the impact of the proposed HW-fingerprint on indoor locations, we calculated the loss rate of the important APs selected to construct the Ratio fingerprint; loss rate was the value of $UDect_{times}^{LR}$ to $ALL_{num}^{LR}$, where $UDect_{times}^{LR}$ means the times that an AP was undetected in all fingerprints collected by all RPs and $ALL_{num}^{LR}$ was the total number of fingerprint samples collected by all RP.s As shown in Figure 11b,c, SVM and CNN had a certain improvement in location-accuracy rate in most cases. Loss rate is shown in Table 6 and, according to Figure 11b,c, when the number of APs is lost and loss rate was high like Days 2, 7, and 16, there was still improvement in location accuracy.

In the experiment, the selected AP was from measurable APs in the experiment environment, and the important APs used to construct the Ratio fingerprint were obtained by analyzing the training data. Therefore, it is inevitable that these important APs were closed in the subsequent time period. When some APs were used to construct the Ratio fingerprint, its RSSI could not be detected, which caused many null values when building the online HW-fingerprint. As a result, the HW-fingerprint built online did not match the HW-fingerprint built in the offline phase because of the addition of these noises.

In Table 7, we calculated the distance error of the three algorithms in the cases of using and not using the HW-fingerprint. Distance error was the average error of their predicted and actual positions about the online HW-fingerprint. It can be seen that positioning error was reduced when the HW-fingerprint was used in the case of using three algorithms.

In Table 8, we outline statistics on the run time of the three algorithms in the cases of using and not using the HW-fingerprint. Since the training strategies of each algorithm are different, it was difficult to evaluate their training time. For example, KNN is an algorithm that does not require training. So we only counted the test time for a single sample. It can be seen that the increase of running time when using HW-fingerprint was negligible and did not have much impact on the servers.

**Figure 12:**
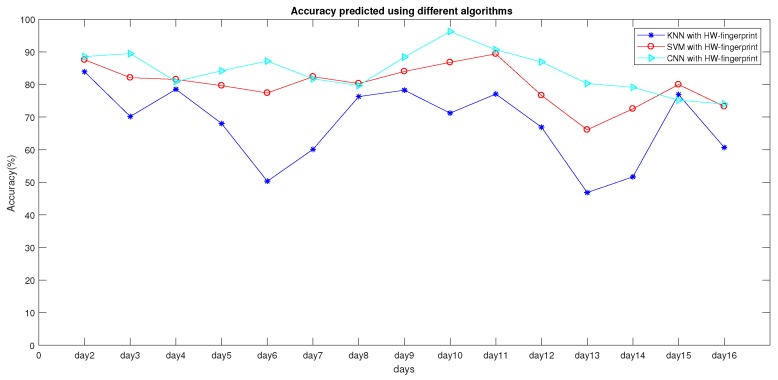
Comparison between experiment results with different algorithms.

Finally, the experiment results of different algorithms are shown in Figure 12 and Table 9 when using the HW-fingerprint. It can be seen that the CNN was significantly better than KNN and SVM. The CNN was 4.19% and 16.37% higher than KNN and SVM in average daily location accuracy, respectively. Compared with SVM and KNN, CNN’s test results showed that the deep-learning model had better performance in data-feature extraction and classification.

### 3.5. Discussion

As shown in the experimental section above, we conducted a series of experiments to establish the feasibility of verifying the proposed method. First, in order to verify the impact of selecting the threshold of the important contribution AP on the quality of the HW-fingerprint, we did a comparative experiment with different thresholds. The experiment results showed that, when the threshold was set to a large value, the HW-fingerprint could achieve better prediction accuracy. Second, in order to verify the influence of different CNN structures on indoor-positioning accuracy, we also carried out related comparison experiments and finally obtained a better CNN positioning model. Finally, we verified the improvement effect of the HW-fingerprint on indoor positioning with different algorithms. Of course, in the prediction of using KNN, when using HW-fingerprint the improvement of prediction accuracy was not so obvious, so we also carried out related analysis. However, in the experiments using SVM and CNN, our proposed HW-fingerprint could significantly improve the accuracy of indoor positioning. This also proved the validity and feasibility of the proposed method.

## 4. Conclusions

In this paper, in order to enhance the ability of fingerprints to express the change characteristics of indoor environments, a feature-construction method based on adding a Ratio fingerprint was proposed. We tested the HW-fingerprint in an actual environment, and the test results showed that, in the case with a HW-fingerprint compared with the case without a HW-fingerprint, the average daily improvement location accuracy of KNN, SVM and CNN increased by 3.39%, 8.03% and 9.03%, respectively. The CNN method was 4.19% and 16.37% higher than SVM and KNN in average daily location accuracy, respectively. The improvement was limited to a few days, and the reason was the AP we chose to build the Ratio fingerprint by statistical methods rather than long-term investigations. Of course, our experiment environment was still not big enough, but in small areas, prediction results are prone to errors due to the close distance between the RPs. In future work, we will consider a larger environment and focus on the work of stable AP selection and methods to increase the positioning performance of the system.

## Figures and Tables

**Table 1:** Weather in experiment environment.

Date	Maximum Temperature	Minimum Temperature	Weather	Humidity
15 November 2018	28 °C	20 °C	Sunny	88%
16 November 2018	27 °C	20 °C	Light rain	96%
17 November 2018	28 °C	21 °C	Light rain	99%
18 November 2018	25 °C	18 °C	Cloudy	98%
19 November 2018	19 °C	11 °C	Cloudy	85%

**Table 2:** Summary of parameters used in deep-learning classifier.

Parameters	Value
Batch size	50 (number of training samples)
Learning rate	0.001
Training epoch	20 (iteration training times)
Dropout rate	0.5

**Table 3:** Accuracy analysis of different thresholds.

Thresholds	Average Accuracy of 15 Days	Variance
0.7	80.00%	0.0066
0.8	81.00%	0.0111
0.9	84.17%	0.0041

**Table 4:** Accuracy analysis of different kernel sizes.

Thresholds	Average Accuracy of 15 Days	Variance
3*3	84.17%	0.0038
5*5	82.83%	0.0041
7*7	78.42%	0.0070

**Table 5:** Average daily location accuracy.

Algorithm	Without HW-Fingerprint	With HW-Fingerprint	Average Improvement Accuracy
KNN	64.39%	67.79%	3.39%
SVM	71.94%	79.97%	8.03%
CNN	75.13%	84.17%	9.03%

**Table 6:** Loss of important APs during the 15 days of testing.

Day	Serial Number of Lost APs	Loss Rate
day2	1,7,8	15.55%, 12.17%, 20.61%
day3	2,8	41.97%, 10.40%
day4	1,9	19.04%, 13.97%
day5	1,7,8	20.98%, 47.84%, 15.77%
day6	12	100%
day7	1,7,8,12	12.26%, 30.50%, 12.26%, 54.79%
day8	1,8	24.29%, 13.93%
day9	1,8	16.62%, 13.87%
day10	1,7	20.08%, 42.15%
day11	1	25.72%
day12	12	31.82%
day13	7,8	55.76%, 14.31%
day14	1,7,8	10.83%, 44.89%, 24.81%
day15	8	10.83%
day16	2,8,10,12	24.00%, 11.39%, 18.25%, 45.14%

**Table 7:** Distance error.

Algorithm	Distance Error without HW-Fingerprint	Distance Error with HW-Fingerprint
KNN	4.6563 m	4.1681 m
SVM	4.2772 m	4.1145 m
CNN	4.3929 m	3.9118 m

**Table 8:** Time to test a single sample.

Algorithm	Running Time without HW-Fingerprint	Running Time with HW-Fingerprint
KNN	0.0008 s	0.0012 s
SVM	0.0020 s	0.0023 s
CNN	0.0008 s	0.0035 s

**Table 9:** Average daily location accuracy.

Algorithm	Average Daily Location Accuracy
KNN	67.79%
SVM	79.97%
CNN	84.17%

## Abbreviations

The following abbreviations are used in this manuscript:

RSSI	Received Signal Strength Indicator
HW-fingerprint	Hybrid Wireless fingerprint
CNN	Convolutional Neural Network
KNN	K-Nearest Neighbor
SVM	Support Vector Machines
Wireless Sensor Network	WSN
DV-Hop	Distance Vector-Hop
MAC	Media Access Control Address
DNN	Deep Neural Network
RNN	Recurrent Neural Network
MAC	Media Access Control Address
RFID	Radio Frequency IDentification
LBS	Location-Based Services
WLANs	Wireless Local Area Networks
AOA	Angle Of Arrival
TOA	Time Of Arrival
HMM	Hidden Markov Model
CSI	Channel State Information
DBN	Deep Belief Networks
CL	Convolutional Layers
FC	Fully Connected
AP	Access Point
RP	Reference Point
ReLu	Rectified Linear Units
